# 17-β-estradiol inhibits hyperosmolarity-induced proinflammatory cytokine elevation via the p38 MAPK pathway in human corneal epithelial cells

**Published:** 2012-05-01

**Authors:** Changjun Wang, Xin Shi, Xiaoyi Chen, Han Wu, Huina Zhang, Jiajun Xie, Xianyan Yang, Zhongru Gou, Juan Ye

**Affiliations:** 1 Department of Ophthalmology, the Second Affiliated Hospital, Zhejiang University School of Medicine, Hangzhou, China; 2 Zhejiang-California International NanoSystems Institute, Zhejiang University, Hangzhou, China

## Abstract

**Purpose:**

To evaluate the effects of 17-β-estradiol on hyperosmolar stress-induced proinflammatory cytokine production of interleukin (IL)-6, IL-1, and tumor necrosis factor-alpha (TNF-α) in SV40-immortalized human corneal epithelial cells (hCECs) and the regulatory effects of the mitogen-activated protein kinase (MAPK) signaling pathways in this process.

**Methods:**

SV40 hCECs cultured in normal osmolar media were switched to a higher osmolarity (450 mOsM) by adding NaCl with or without pretreatment with 17-β-estradiol. Real-time polymerase chain reaction and ELISA were applied to characterize IL-6, IL-1, and TNF-α gene and protein expression. Cells were treated for 15−60 min, lysed in radioimmunoprecipitation assay (RIPA) buffer and subjected to a western blot with phospho (p)-specific antibodies against extracellular signal-regulated protein kinase 1/2 (ERK1/2), P38 kinase, and c-Jun N-terminal kinase 1/2 (JNK1/2).

**Results:**

The expression and production of IL-6, IL-1, and TNF-α in SV40 hCECs increased when the media osmolarity was switched to 450 mOsM. Pretreatment with 10^−10^ M 17-β-estradiol greatly inhibited the increased expression and production of IL-6, IL-1, and TNF-α induced by hyperosmolarity, whereas with the administration of SB203580 (10 μM), an inhibitor of the p38 pathway, the inhibiting effect of 17-β-estradiol disappeared. The western blot results showed that the increased phosphorylation level of p38 caused by hyperosmolarity was greatly inhibited by 17-β-estradiol.

**Conclusions:**

17-β-estradiol greatly inhibited the expression and production of proinflammatory cytokines IL-6, IL-1, and TNF-α, which were stimulated by hyperosmolarity in SV40-immortalized hCECs. The results also suggested that the p38 MAPK signaling pathway was involved in the regulatory effects of estrogen on hCECs. These findings may contribute to an understanding of the etiologic roles and therapeutic implications of the hormone estrogen in dry eye disease.

## Introduction

Dry eye disease (DED) is among the most common and problematic conditions confronted by ophthalmologists. Most epidemiologic studies have demonstrated a higher prevalence of dry eye syndrome in the elderly, especially in postmenopausal women [1-3]. The significant gender-based differences in the incidence of dry eyes suggest that estrogens play an important role in DED. Although many studies have produced evidence of estrogen receptors in ocular surface tissues [4,5], the mechanism by which estrogens influence the ocular surface is not yet clear. Jensen et al. [6] reported that hormone replacement therapy (HRT) in postmenopausal women may help alleviate symptoms related to ocular dryness. However, a randomized trial showed no strong evidence to support the use of HRT for treating dry eye syndrome [7], and Schaumberg et al. [8] suggested that women who use HRT, particularly estrogen alone, are at an increased risk of DED. Such controversies regarding the risks and benefits of estrogen therapy on dry eyes highlights the complex role of sex hormones in ocular surface health and the need for further study.

Recent studies have suggested that hyperosmolarity is a key factor in the pathogenesis and diagnosis of DED. The inadequate secretion of tears and increased tear evaporation, either of which results in hyperosmolarity, are two major causes of DED [9]. The normal osmolarity of the tear fluid is approximately 300 mOsm/kg [10] and the suggested “gold standard” referent for the diagnosis of DED is 316 mOsm/kg or greater [11,12]. Two common mechanisms contributing to the pathogenesis of ocular surface injury in DED are tear hyperosmolarity and ocular surface inflammation [13]. There is increasing evidence that tear hyperosmolarity triggers ocular surface inflammation cascades [14]. Liu et al. [15] also reported that people subjected to the instillation of NaCl and sucrose hyperosmolar drops complained of dry eye discomfort. Furthermore, hyperosmolar media-cultured hCECs have been widely used to study the pathogenesis of and therapeutic interventions that may alleviate DED [16-18].

A previous research has investigated the effects that estrogen may have on the corneal epithelial cells in cases of DED [19]. However, much of this work was performed in an isosmotic environment that did not perfectly simulate the pathological changes on the cornea in cases of DED. Furthermore, the previous study rarely examined the signaling pathways to clarify the underlying mechanisms. Therefore, the purpose of the current study was to investigate the effects of estrogen on hyperosmolarity-stressed hCECs and to elucidate the regulatory effects that the mitogen-activated protein kinase (MAPK) signaling pathways may have involved.

## Methods

### Material and reagents

Cell culture dishes, plates, centrifuge tubes, and other plastic wares were purchased from BD Bioscience (Lincoln Park, NJ). DMEM/Ham’s F12 medium (50% Dulbecco Modified Eagle Medium and 50% Ham’s Nutrient Mixture F-12), phosphate buffered saline (PBS), 0.25% trypsin, DNA or RNA size markers, and a random primer DNA labeling kit were purchased from Invitrogen Gibco (Grand Island [GIBCO], NY). Fetal bovine serum (FBS) was purchased from Hyclone (Logan, UT). Sodium chloride (NaCl), 17-β-estradiol, dimethyl sulfoxide (DMSO), ethanol, human recombinant EGF, and all other reagents were purchased from Sigma-Aldrich (St. Louis, MO). Recombinant human interleukin (IL)-6, IL-1, and tumor necrosis factor-alpha (TNF-α) enzyme-linked immunosorbent assay (ELISA) kits (eBioscience, San Diego, CA) were used. The other materials used were phospho-specific monoclonal antibodies for extracellular signal-regulated protein kinase (ERK), c-Jun N-terminal kinase (JNK), and p38 kinase and rabbit antibodies from Santa Cruz Biotechnology (Santa Cruz, CA); nitrocellulose membranes from Schleicher and Schuell (Keene, NH); SYBR Premix Ex Taq, Polymerase Chain Reaction (PCR) forward primer, PCR reverse primer, ROX reference dye and DNA template from TaKaRa BIO, Inc. (Shiga, Japan).

### Culture of hCECs

The Simian virus (SV) 40-immortalized hCECs were kindly provided by Dr. Zan Pan (State University of New York, New York, NY) and cultured in DMEM/F12, which was supplemented with 10% fetal bovine serum and 10 ng/ml human recombinant epidermal growth factor. Cells were incubated in a CO_2_-regulated incubator in a humidified 95% air/5% CO_2_ atmosphere. The medium was renewed every two days. After the culture reached confluence, cells were detached with 0.25% trypsin and seeded into 96-well and 24-well plates at a density of 5×10^3^ and 5×10^5^ cells/well, respectively.

### Cell viability assay

After implantation into 96-well plates for 24 h, cells were placed in serum-free medium. 17-β-estradiol was added at final concentrations of 10^−8^, 10^−9^, 10^−10^, and 10^−11^ M. Both controls without 17-β-estradiol, and 17-β-estradiol-treated cells were incubated for an additional 24 h. Subsequently, cells were collected and resuspended with 1× assay buffer at a cell density of 2×10^5^/ml. One hundred μl of the cell suspension was labeled with Annex-V/propidium iodide (PI) and analyzed by using flow cytometry (The Becton Dickinson LSR flow cytometer; Becton Dickinson, Franklin Lakes, CA).

### RNA extraction and real-time PCR

Cells were seeded in 24-well plates at a density of 5×10^5^ cells/well. After 24 h, the medium was replaced with a serum-free DMEM/F12 medium. According to the method of Luo et al. [20], hyperosmolarity (450 mOsM) was achieved by adding NaCl to reach a final concentration of 70 mM. Cells were exposed to the hyperosmotic medium pretreated with 10^−10^ M 17-β-estradiol for 40 min. Cells were then collected at different time points for the measurement of *IL-6*, *IL-1*, and *TNF-α* expression. Total cellular RNA was isolated by using TRIzol reagent (Invitrogen, Carlsbad, CA). The RNA was treated with Deoxyribonuclease I (Invitrogen) to remove the chromosomal DNA, evaluated spectrophotometrically at 260 nm to determine the concentration, and examined on 1.0% agarose (GIBCO) gels to confirm RNA integrity. Real-time PCR was used to confirm the differential expression of the *IL-6*, *IL-1*, and *TNF-α* genes. Cells cultured in a hyperosmotic medium without any 17-β-estradiol treatment were used as controls. Sense and antisense primers were designed using Primer Express Software, version 1.0 (Applied Biosystems, Inc., Foster City, CA; Table 1). Real-time PCRs were performed according to the manufacturer’s recommendations, using 10 μl SYBR Premix Ex Taq (2×), 0.4 μl PCR forward primer (10 μM), 0.4 μl PCR reverse primer (10 μM), 0.4 μl ROX reference dye II (50×), 2 μl DNA template, 6.8 μl ddH2O in 96-well reaction plates, ABI PRISM optical adhesive covers, and the GeneAmp 7500 sequence detection system. The amplification program involved 30 s at 95 °C, followed by 40 cycles of 15 s at 95 °C and 3 s at 60 °C. The relative gene expression was determined by using the 7500 software V2.0.1 (Applied Biosystems [ABI], Foster City, CA) and standardizing the levels to those of β-actin (*ACTB*) mRNA. Dissociation curves were evaluated to ensure the absence of secondary PCR products.

**Table 1:** Oligonucleotide primers designed for real-time PCR.

**Primer**	**Orientation**	**Nucleotide sequence (5′-3′)**	**Amplicon size (bp)**
*IL-1*	Sense	TGGCGGCATCCAGCTACGAA	247
	Antisense	CCGGAGCGTGCAGTTCAGTGA	
*IL-6*	Sense	AGCGCCTTCGGTCCAGTTGC	240
	Antisense	TGCCAGTGCCTCTTTGCTGCT	
*TNF-α*	Sense	TTCCTGATCGTGGCAGGCGC	246
	Antisense	CAGCTCCACGCCATTGGCCA	
*ACTB*	Sense	CGTTGACATCCGTAAAGACC	242
	Antisense	AACAGTCCGCCTAGAAGCAC	

### IL-6, IL-1, and TNF-α measurements

Cells were seeded in 24-well plates at a density of 5×10^5^ cells. After 24 h, the medium was replaced with a serum-free DMEM/F12 medium. Hyperosmolarity (450 mOsM) was achieved by adding NaCl to reach a final concentration of 70 mM. Cells were exposed to the hyperosmotic medium, which was pretreated with 10^−10^ M 17-β-estradiol with or without SP600125 50 μM (an inhibitor of the JNK pathway), SB203580 10 μM (an inhibitor of the p38 pathway), or U0126 10 μM (an inhibitor of the ERK pathway) for 40 min. The culture medium was then collected for the measurement of IL-6, IL-1, and TNF-α and compared with cells cultured in media that had not been pretreated with 17-β-estradiol. All the collected media were centrifuged at 800× g for 5 min, and the supernatants were transferred to vials and stored at −80 °C until the various cytokines were measured. The concentrations of IL-6, IL-1, and TNF-α were measured in triplicate by using recombinant human IL-6, IL-1, and TNF-α ELISA kits in accordance with the manufacturer’s instructions. Amounts were expressed in picograms per milliliter (pg/ml).

### Western blot analysis

The cells treated for 15–60 min were lysed in RIPA buffer, and appropriate volumes (20–30 μl) of cell extracts, adjusted to represent the same amount of total cellular protein (50 μg), were electrophoresed under reducing conditions at 4 °C in a 4% to 15% gradient polyacrylamide gel. After electrophoretic transfer to a nitrocellulose (NC) membrane at 100 V for 1 h at 4 °C, the membranes were blocked with 10% nonfat milk in TBST (10mM Tris-HCl, pH7.5, 150mM NaCl, 0.05%Tween-20) for 4 h at room temperature. The primary antibody (i.e., phospho-specific antibodies against JNKs, ERKs, or p38) in TBST containing 10% nonfat milk was placed on each membrane and incubated at room temperature for 3 h. After three washings with TBST over 15 min, the second antibody, conjugated with horseradish peroxidase (HRP) was applied. After three washings with TBST over 15 min, the membrane was analyzed by the Odyssey® Infrared Imaging System (LI-COR Biosciences, Lincoln, NE).

### Statistical analysis

Statistical comparisons of control and treatment groups for real-time PCR and ELISA data were performed via One-way ANOVA (statistical package SPSS10.0 [SPSS Inc., Chicago, IL]), with p<0.05 considered to be statistically significant.

## Results

### Cell viability assay

**Figure 1:**
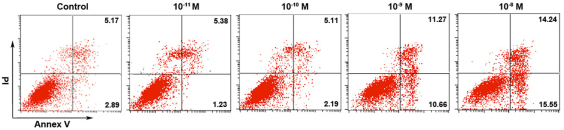
Effects of 17-β-estradiol on cell viability. Cells were cultured with 17-β-estradiol at different concentrations for 24 h. The percentage of apoptotic cells were calculated (10^−8^ M 14.24%; 10^−9^ M 11.27%; 10^−10^ M 5.11%; 10^−11^ M 5.38%, respectively). Statistical analysis showed that 17-β-estradiol at concentrations of 10^−10^ and 10^−11^ M did not show a significant difference (p>0.05) as compared with the controls, cells cultured without 17-β-estradiol.

Flow cytometry (Figure 1) showed that cultured hCECs treated with 17-β-estradiol at concentrations of 10^−10^ M and 10^−11^ M for 24 h did not show a significant difference in cell viability as compared with cells cultured without 17-β-estradiol (Figure 1; control). With the use of 17-β-estradiol at concentrations of 10^−8^ M and 10^−9^ M, the cell apoptosis percentages were increased to 14.24% and 11.27%, respectively, which suggested that treatment with 17-β-estradiol at concentrations higher than 10^−10^ M may have adverse effects on cell survival. Therefore, we used 10^−10^ M 17-β-estradiol in all of the following experiments in this study.

### Effects of estrogen on IL-6, IL-1, and TNF-α mRNA expression

**Figure 2:**
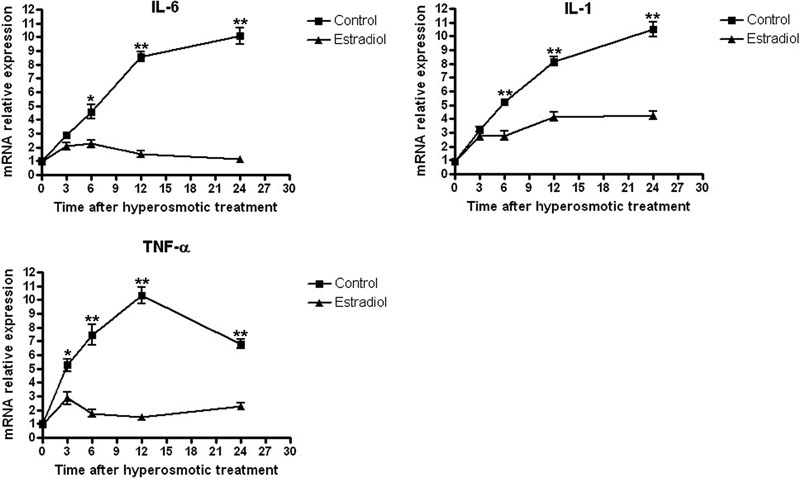
Real-time PCR results of *IL-6*, *IL-1*, and *TNF-α* mRNAs. Real-time PCR results of *IL-6*, *IL-1*, and *TNF-α* mRNAs in SV40-immortalized HCECs pretreated with 17-β-estradiol and exposed to hyperosmolar media for 24 h. Controls were cells cultured in hyperosmolar media without pretreatment with 17-β-estradiol. *IL-6*, *IL-1*, and *TNF-α* mRNA expression was gradually upregulated in a 450 mOsM hyperosmotic medium from 3 to 24 h. Pretreatment with 17-β-estradiol greatly inhibited *IL-6*, *IL-1*, and *TNF-α* mRNA expression and the suppression effect lasted as long as 24 h. Similar results were obtained in three independent experiments. *p<0.05, **p<0.01.

Real-time PCR analysis revealed that *IL-6*, *IL-1*, and *TNF-α* mRNA expression was gradually upregulated in a 450 mOsM hyperosmotic medium as exposure time was prolonged (3−24 h) (Figure 2). The pretreatment with 17-β-estradiol for 40 min greatly inhibited *IL-6*, *IL-1*, and *TNF-α* mRNA expression in the early stage of the hyperosmotic stimulation, and the suppression effect lasted as long as 24 h. The inhibitory effects of 17-β-estradiol on *IL-6*, *IL-1*, and *TNF-α* mRNA expression increased with time and at 24 h, the suppressive effects reached 90.1%, 60.9%, and 68.5%, respectively.

### Estrogen reduced hyperosmolarity-induced IL-6, IL-1, and TNF-α production

**Figure 3:**
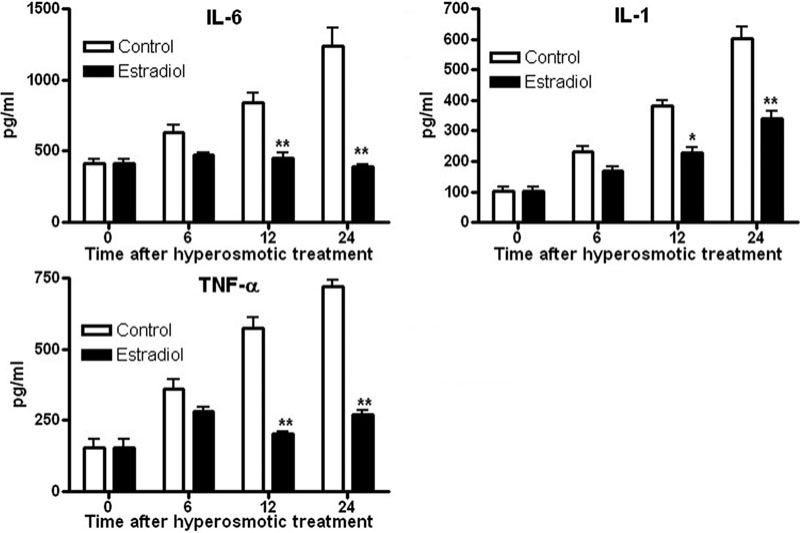
ELISA results regarding the amounts of IL-6, IL-1, and TNF-α in HCECs. Pretreatment with 17-β-estradiol inhibited the hyperosmolarity- induced increases in IL-6, IL-1, and TNF-α production. Data are the mean±SEM of the results from three independent experiments. With pretreatment with 17-β-estradiol, IL-6, IL-1, and TNF-α production was greatly inhibited (black box) as compared with cells cultured in hyperosmolar media without pretreated 17-β-estradiol (white box). *p<0.05, **p<0.01.

ELISA was used to determine the effects of the 40-min pretreatment with 10^−10^ M 17-β-estradiol on the hyperosmolarity-induced increases in IL-6, IL-1, and TNF-α production. At 24 h, IL-6, IL-1, and TNF-α production was about 2–4 fold greater than that of the control group, without the 17-β-estradiol pretreatment. At 24 h, IL-6, IL-1, and TNF-α protein in pretreated cells were as much as 68.65%, 43.68%, and 62.73% less, respectively, than in cells that had not been pretreated with 17-β-estradiol (Figure 3). That is, comparing the two groups, these proteins were present in a smaller amount in the pretreated cells than in the non-pretreated cells. These results indicated that IL-6, IL-1, and TNF-α production was greatly inhibited in the 17-β-estradiol-pretreated cells.

### Effects of estrogen on the activation of the MAPK pathway

**Figure 4:**
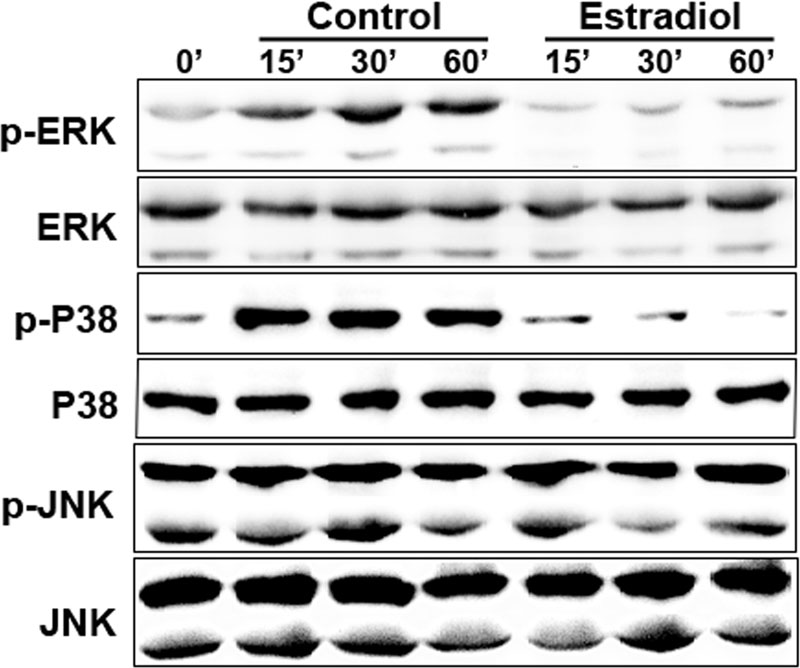
Western blot results of HCECs cultured in 450 mOsM hyperosmolar media. The western blot shows the time course of activated phosphorylated p38, which is apparently inhibited by 17-β-estradiol in SV40-immortalized human corneal epithelial cells cultured in 450 mOsM hyperosmolar media.

**Figure 5:**
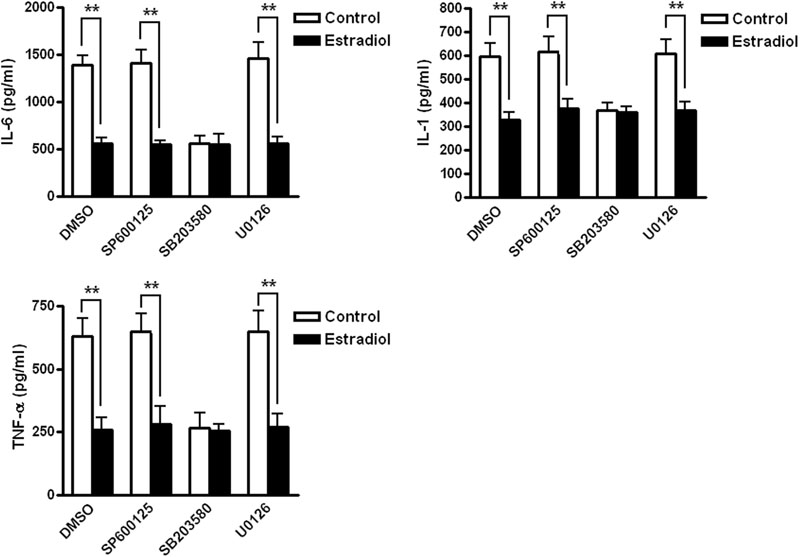
ELISA results regarding the amounts of IL-6, IL-1, and TNF-α present with inhibitors of the MAPK pathway in HCECs. Inhibitors of the JNK pathway (SP600125 50 μM), the p38 pathway (SB203580 10 μM), and the ERK pathway (U0126 10 μM) were used, but only SB203580 (an inhibitor of the p38 pathway) suppressed the elevation of cytokines caused by hyperosmolarity. 17-β-estradiol suppressed the hyperosmoticity-induced elevation of all of the three cytokines in conditions with or without JNK/ERK/p38 MAPK inhibitors. **p<0.01.

Using phosphor-specific antibodies, western blot analysis revealed that the activated (phosphorylated) p38 was dramatically inhibited by pretreatment with 17-β-estradiol in SV40-immortalized hCECs within 15−60 min of exposure to 450 mOsM hyperosmolar media. In the control group, stimulated by the hyperosmolarity, p38 began to activate at the 15 min time point, and the maximum activation was observed after 30 min. A similar response was also observed in the case of p-ERK, with an increase by 15 min and the maximal activation being reached at 30 min. In the group of cells, which were pretreated with 17-β-estradiol, p38, and ERK were rarely activated by the hyperosmolarity (Figure 4). However, there was no difference in p-JNK between the pretreated cells and the controls. When inhibitors of the JNK pathway (SP600125 50 μM), the p38 pathway (SB203580 10 μM), and the ERK pathway (U0126 10 μM) were used, only the p38 inhibitor SB203580 could suppress the elevation of cytokines caused by hyperosmolarity (Figure 5). Figure 5 also showed that 17-β-estradiol pretreatment could suppress hyperosmotic-induced increases in any of the cytokines in all conditions with or without JNK/ERK/p38 MAPK inhibitors. Because all three inhibitors of the pathways were dissolved in DMSO, we used DMSO as a negative control.

## Discussion

This study demonstrated that 17-β-estradiol inhibited the expression of proinflammatory cytokines in immortalized hCECs. 17-β-estradiol pretreatment caused a significant decrease in the levels of *IL-6*, *IL-1*, and *TNF-α* mRNA as compared with hyperosmolar media-exposed control cells. The ELISA data provided further support for the notion that 17-β-estradiol suppresses the production of IL-6, IL-1, and TNF-α induced by hyperosmolarity at the protein level. Both of the results showed that the expression of IL-6, IL-1, and TNF-α remained at a constant low level throughout the entire course of the experiment. In contrast, the IL-6, IL-1, and TNF-α levels in the control group were dramatically increased with time. These results indicated that the efficacy of 17-β-estradiol could be maintained for an extended period. Furthermore, of the three inhibitors added to the culture media (SP600125 50 μM for the JNK pathway, SB203580 10 μM for the p38 pathway, and U0126 10 μM for the ERK pathway), SP600125 and U0126 did not inhibit the effect of the hyperosmolarity-induced elevation of cytokines in cells. Only p38 inhibitor SB203580 could suppress the elevation of cytokines caused by hyperosmolarity.

There is growing recognition that inflammation plays an important role in DED, and increased levels of proinflammatory cytokines and markers have been observed in both the tear films and ocular surface epithelia of patients with dry eye [21,22]. TNF-α, IL-1, and IL-6 are cytokines that have been the focus of various studies on DED [23-25]. In our present study, both the mRNA expression and the protein production of the three cytokines showed dramatic increases in response to a hyperosmolar stimulus.

The ability of estrogen to inhibit proinflammatory cytokines is analogous to the hormone’s action in other sites of the human body. Elisabetta et al. [26] reported that estrogen prevented lipopolysaccharide-induced inflammatory response in microglia in the central nervous system, demonstrating a protective role of estrogen in neurodegenerative disorders in humans and experimental animal models. Other evidence has indicated that estradiol supplementation reduced the production of TNF-α, IL-1β, and IL-6 and decreased the activation of p38 MAPK as compared to their untreated counterparts, which suggested that the effect of estrogen on cardioprotection may be attributed to its anti-inflammatory properties [27]. As in our study, similar MAPK changes were observed in the context of estrogen protection against hyperosmolarity-induced proinflammatory cytokine elevation in hCECs, i.e., proinflammatory cytokine production was inhibited via the p38 MAPK signaling pathways. The MAPK signaling pathways play an important role in the cellular response to specific stimuli, and they also regulate specific substrates [28,29]. MAPKs include JNK, p38 and ERK. ERK is mainly involved in cell survival, cell proliferation and hyperosmolar stress [30], while p38 and JNK cascades are strongly activated by various extracellular stimuli, including changes in osmolarity [31,32]. It is certain that the MAPK pathways were involved in the elevation of hyperosmolarity-induced proinflammatory cytokines in hCECs, but which special pathway of the MAPK pathways mediates increases in cytokine release during exposure to hypertonicity is yet unclear. Different literatures have stated that different MAPK pathways take part in mediating the increases in cytokine release during exposure to hypertonicity. Hyperosmolarity’s effects on the activation of MAPK are highly cell-specific. Selective or combined activation of ERK, p38 and/or JNK by hyperosmoticity stimulation depends on the cell type [33].

Using phosphor-specific antibodies, western blot analysis revealed that the phosphorylated p38 and ERK were dramatically activated by hyperosmolar stress and that the phosphorylation of p38 and ERK was greatly inhibited by pretreatment with 17-β-estradiol. Our purpose of this research project was to investigate the specific pathway involved in the elevation of proinflammatory cytokine. When the inhibitors of the ERK pathway (U0126 10 μM) and the p38 pathway (SB203580 10 μM) were used, only the p38 inhibitor SB203580 could suppress the elevation of proinflammatory cytokines caused by hyperosmolarity. The result of our study demonstrated that 17-β-estradiol can suppress hyperosmotic-induced increases in any of the cytokines in all conditions with or without JNK/ERK/p38 MAPK inhibitors, while in the absence of p38 MAPK activity caused by exposure to SB203580, the effects of hyperosmotic-induced increases in any of the cytokines were suppressed, which suggested that the p38 MAPK pathway was involved in the elevation of hyperosmolarity-induced proinflammatory cytokines in hCECs. It also indicated that 17-β-estradiol had a somewhat similar role in the effect of hyperosmotic-induced increases in the cytokines as that of the p38 MAPK inhibitor. Chen et al. found that the activation of p38MAP kinase (p38), JNK MAP kinase (JNK) and NF-kB in cultured corneal epithelial cells was induced by hyperosmotic conditions [13]. Furthermore, they found that p38 inhibitor significantly suppressed hyperosmoticity-induced IL-1b production, whereas JNK inhibitor failed to do so. Therefore, they postulated that hyperosmoticity-induced IL-1b expression was mainly achieved via the p38 pathway in hCECs. The situation in our study is to some extent similar to the results reported by Chen et al. [13]. The western blot showed that both p-p38 and p-ERK were greatly suppressed over the time course and ELISA results showed that inhibiting the ERK pathway (with U0126 10 μM) did not influence the level of cytokines. It demonstrated that the ERK signaling pathway may be activated by the hyperosmolar stimulus and that it could also be suppressed by the 17-β-estradiol, but this ERK pathway was not involved in the regulatory effect of estrogen in the production of proinflammatory cytokines. The ERK signaling pathway probably participates in some other aspects of the interactions between estradiol and hyperosmolarity-stressed hCECs.

It is important to note that our results relate to 17-β-estradiol and the expression of corneal proinflammatory cytokines, such as IL-6, IL-1, and TNF-α, in immortalized cells. Whether such estrogen actions also occur in primary corneal epithelial cells in vitro or in vivo and are associated with parallel alterations in the protein and signaling pathways remains to be determined. However, we processed the cells in hyperosmolar media, which could mimic the pathological changes in DED in vivo to a large extent. Many studies have suggested that hyperosmolarity is a key factor in the pathogenesis and diagnosis of DED. We used 450 mOsM osmolarity in our experiment because this osmolarity is the most commonly used osmolarity in dry eye research [17,20,29]. Tear osmolarity in dry eye patients ranges from 306 to 441 mOsm, with an average of 343 mOsm. However, a recent study suggests that transiently, tear hyperosmolarity may be much higher when local tear film thinning or breakup occurs [34]. Therefore, the osmolarity of 450 mOsM could well reflect the hyperosmolarity-induced proinflammatory cytokine elevation in human corneal pathogenesis. However, Suzuki et al. [19] reported that 17-beta-estradiol induced proinflammatory cytokines in immortalized hCEC. The reason for the discrepancy may be that the previous work was done in normal condition while our present work was done in hyperosmolar condition. Under stressful conditions, cells were forced to adapt for survival. When cells were cultured in hyperosmolar condition, different kinds of protein and gene expressions might occur, which resulted in divergent reactions to the stimulus.

Although the role of estrogen in dry eye has been recognized and the pathologic mechanisms in dry eyes are being investigated, minimal data are available regarding the effect of pregnancy, hysterectomy and androgens on the ocular surface. The complex role of sex hormones on ocular surface health and disease warrants further study. Postmenopausal women are known to have an increased risk of dry eyes. Lactation (low estrogen state) is also associated with an increased risk of dry eyes. While pregnancy, oral contraceptive use, and hormone replacement (a high estrogen state) also result in dry eyes, these conflicting findings indicated that there may be a balance among different hormones. A thorough understanding of this will help us to make appropriate guidelines for hormone replacement therapy to alleviate hyperosmolarity-induced corneal inflammatory responses in post-menopausal women.

In conclusion, 17-β-estradiol greatly inhibited the expression and production of proinflammatory cytokines IL-6, IL-1, and TNF-α, which were stimulated by hyperosmolarity via the p38 MAPK signaling pathway in SV40-immortalized hCECs. These findings may contribute to the understanding of the etiologic roles and therapeutic implications of the hormone estrogen in DED.

## References

[1] Gayton JL (2009). Etiology, prevalence, and treatment of dry eye disease.. Clin Ophthalmol.

[2] Schaumberg DA, Sullivan DA, Buring JE, Dana MR (2003). Prevalence of dry eye syndrome among US women.. Am J Ophthalmol.

[3] Schaumberg DA, Dana R, Buring JE, Sullivan DA (2009). Prevalence of dry eye disease among US men: estimates from the Physicians' Health Studies.. Arch Ophthalmol.

[4] Wickham LA, Gao J, Toda I, Rocha EM, Ono M, Sullivan DA (2000). Identification of androgen, estrogen and progesterone receptor mRNAs in the eye.. Acta Ophthalmol Scand.

[5] Suzuki T, Kinoshita Y, Tachibana M, Matsushima Y, Kobayashi Y, Adachi W, Sotozono C, Kinoshita S (2001). Expression of sex steroid hormone receptors in human cornea.. Curr Eye Res.

[6] Jensen AA, Higginbotham EJ, Guzinski GM, Davis IL, Ellish NJ (2000). A survey of ocular complaints in postmenopausal women.. J Assoc Acad Minor Phys.

[7] Piwkumsribonruang N, Somboonporn W, Luanratanakorn P, Kaewrudee S, Tharnprisan P, Soontrapa S (2010). Effectiveness of hormone therapy for treating dry eye syndrome in postmenopausal women: a randomized trial.. J Med Assoc Thai.

[8] Schaumberg DA, Buring JE, Sullivan DA, Dana MR (2001). Hormone Replacement Therapy and Dry Eye Syndrome.. JAMA.

[9] (2007). The Definition and Classification of Dry Eye Disease: Report of the Definition and Classification Subcommittee of the International Dry Eye WorkShop.. Ocul Surf.

[10] Murube J (2006). Tear Osmolarity.. Ocul Surf.

[11] Tomlinson A, Khanal S, Ramaesh K, Diaper C, McFadyen A (2006). Tear Film Osmolarity: Determination of a Referent for Dry Eye Diagnosis.. Invest Ophthalmol Vis Sci.

[12] Benelli U, Nardi M, Posarelli C, Albert TG (2010). Tear osmolarity measurement using the TearLab Osmolarity System in the assessment of dry eye treatment effectiveness.. Cont Lens Anterior Eye.

[13] Chen M, Hu DN, Pan Z, Lu CW, Xue CY, Aass I (2010). Curcumin protects against hyperosmoticity-induced IL-1beta elevation in human corneal epithelial cell via MAPK pathways.. Exp Eye Res.

[14] Luo L, Li DQ, Corrales RM, Pflugfelder SC (2005). Hyperosmolar saline is a proinflammatory stress on the mouse ocular surface.. Eye Contact Lens.

[15] Liu H, Begley C, Chen M, Bonanno J, McNamara NA, Nelson JD, Simpson T (2009). A link between tear instability and hyperosmolarity in dry eye.. Invest Ophthalmol Vis Sci.

[16] Li DQ, Chen Z, Song XJ, Luo L, Pflugfelder SC (2004). Stimulation of matrix metalloproteinases by hyperosmolarity via a JNK pathway in human corneal epithelial cells.. Invest Ophthalmol Vis Sci.

[17] Chen Z, Tong L, Li Z, Yoon KC, Qi H, Farley W, Li DQ, Pflugfelder SC (2008). Hyperosmolarity-induced cornification of human corneal epithelial cells is regulated by JNK MAPK.. Invest Ophthalmol Vis Sci.

[18] Cavet ME, Harrington KL, Ward KW, Zhang JZ (2010). Mapracorat, a novel selective glucocorticoid receptor agonist, inhibits hyperosmolar-induced cytokine release and MAPK pathways in human corneal epithelial cells.. Mol Vis.

[19] Suzuki T, Sullivan DA (2005). Estrogen stimulation of proinflammatory cytokine and matrix metalloproteinase gene expression in human corneal epithelial cells.. Cornea.

[20] Luo L, Li DQ, Pflugfelder SC (2007). Hyperosmolarity-induced apoptosis in human corneal epithelial cells is mediated by cytochrome c and MAPK pathways.. Cornea.

[21] Narayanan S, Miller WL, McDermott AM (2006). Conjunctival cytokine expression in symptomatic moderate dry eye subjects.. Invest Ophthalmol Vis Sci.

[22] Brignole F, Pisella PJ, Goldschild M, De Saint Jean M, Goguel A, Baudouin C (2000). Flow cytometric analysis of inflammatory markers in conjunctival epithelial cells of patients with dry eyes.. Invest Ophthalmol Vis Sci.

[23] Solomon A, Dursun D, Liu Z, Xie Y, Macri A, Pflugfelder SC (2001). Pro- and Anti-inflammatory Forms of Interleukin-1 in the Tear Fluid and Conjunctiva of Patients with Dry-Eye Disease.. Invest Ophthalmol Vis Sci.

[24] Zoukhri D, Hodges RR, Byon D, Kublin CL (2002). Role of proinflammatory cytokines in the impaired lacrimation associated with autoimmune xerophthalmia.. Invest Ophthalmol Vis Sci.

[25] De Paiva CS, Corralesa RM, Villarreala AL, Farley WJ, Li DQ, Stern ME, Pflugfelder SC (2006). Corticosteroid and doxycycline suppress MMP-9 and inflammatory cytokine expression, MAPK activation in the corneal epithelium in experimental dry eye.. Exp Eye Res.

[26] Vegeto E, Bonincontro C, Pollio G, Sala A, Viappiani S, Nardi F, Brusadelli A, Viviani B, Ciana P, Maggi A (2001). Estrogen Prevents the Lipopolysaccharide-Induced Inflammatory Response in Microglia.. J Neurosci.

[27] Wang M, Tsai BM, Reiger KM, Brown JW, Meldrum DR (2006). 17-β-Estradiol decreases p38 MAPK-mediated myocardial inflammation and dysfunction following acute ischemia.. J Mol Cell Cardiol.

[28] Hubert A, Cauliez B, Chedeville A, Husson A, Lavoinne A (2004). Osmotic stress, a proinflammatory signal in Caco-2 cells.. Biochimie.

[29] Li DQ, Luo L, Chen Z, Kim HS, Song XJ, Pflugfelder SC (2006). JNK and ERK MAP kinases mediate induction of IL-1beta, TNF-alpha and IL-8 following hyperosmolar stress in human limbal epithelial cells.. Exp Eye Res.

[30] Abe J, Kusuhara M, Ulevitch RJ, Berk BC, Lee JD (1996). Big mitogen-activated protein kinase 1 (BMK1) is a redox-sensitive kinase.. J Biol Chem.

[31] Clermont F, Adam E, Dumont JE, Robaye B (2003). Survival pathways regulating the apoptosis induced by tumour necrosis factor-alpha in primary cultured bovine endothelial cells.. Cell Signal.

[32] Pan Z, Capó-Aponte JE, Zhang F, Wang Z, Pokorny KS, Reinach PS (2007). Differential dependence of regulatory volume decrease behavior in rabbit corneal epithelial cells on MAPK superfamily activation.. Exp Eye Res.

[33] Niswander JM, Dokas LA (2007). Hyperosmotic stress-induced caspase-3 activation is mediated by p38 MAPK in the hippocampus.. Brain Res.

[34] Cavet ME, Harrington KL, Ward KW, Zhang JZ (2010). Mapracorat, a novel selective glucocorticoid receptor agonist, inhibits hyperosmolar-induced cytokine release and MAPK pathways in human corneal epithelial cells.. Mol Vis.

